# A Ferroelectric-Photovoltaic Effect in SbSI Nanowires

**DOI:** 10.3390/nano9040580

**Published:** 2019-04-09

**Authors:** Krystian Mistewicz, Marian Nowak, Danuta Stróż

**Affiliations:** 1 Institute of Physics—Center for Science and Education, Silesian University of Technology, Krasińskiego 8, 40-019 Katowice, Poland; marian.nowak@polsl.pl; 2 Institute of Material Science, University of Silesia, 75 Pułku Piechoty 1A, 41-500 Chorzów, Poland; danuta.stroz@us.edu.pl

**Keywords:** antimony sulfoiodide (SbSI), ferroelectric, nanowires, photovoltaic effect, nanodevices

## Abstract

A ferroelectric-photovoltaic effect in nanowires of antimony sulfoiodide (SbSI) is presented for the first time. Sonochemically prepared SbSI nanowires have been characterized using high-resolution transmission electron microscopy (HRTEM) and optical diffuse reflection spectroscopy (DRS). The temperature dependences of electrical properties of the fabricated SbSI nanowires have been investigated too. The indirect forbidden energy gap *E*_gIf_ = 1.862 (1) eV and Curie temperature *T*_C_ = 291 (2) K of SbSI nanowires have been determined. Aligned SbSI nanowires have been deposited in an electric field between Pt electrodes on alumina substrate. The photoelectrical response of such a prepared ferroelectric-photovoltaic (FE-PV) device can be switched using a poling electric field and depends on light intensity. The photovoltage, generated under λ = 488 nm illumination of *P*_opt_ = 127 mW/cm^2^ optical power density, has reached *U*_OC_ = 0.119 (2) V. The presented SbSI FE-PV device is promising for solar energy harvesting as well as for application in non-volatile memories based on the photovoltaic effect.

## 1. Introduction

Ferroelectric nanostructures [1] have been studied with increasing intensity in recent years. They exhibit a wide spectrum of outstanding properties, among others, ferroelectric photovoltaicity, pyroelectricity, high non-linear optical activity, ferroelasticity, and direct and inverse piezoelectricity. Due to this unique combination of different properties, ferroelectric nanomaterials are attractive for application in sensors of mechanothermal signals [2], pyroelectric harvesters for waste heat recovery [3], piezoelectric nanogenerators [4,5], non-volatile memories [6], gas sensors [7,8], photodetectors [2,9], and solar cells [10,11,12].

The ferroelectric-photovoltaic (FE-PV) effect refers to generation of a steady photovoltaic response (photocurrent or photovoltage) along the polarization direction in a ferroelectric material without central symmetry. This phenomenon is distinctly different from the conventional photovoltaic effect in the semiconductor p–n junction, where an internal built-in electric field at the interface of two doped semiconductors separates photo-generated charge carriers. One of the unique properties of FE-PV devices is the possibility to switch photocurrent direction by changing the spontaneous polarization direction of a ferroelectric material with an external electric field. The origin of photovoltage, generated in the FE-PV device, is usually explained taking into account bulk photovoltaic effect [10,13,14,15], domain wall theory [16,17,18], Schottky-junction effect [10,19,20], change of injection barriers, and depolarization electric field [6,10,21,22].

Up till now, a ferroelectric-photovoltaic effect has been intensively studied in bulk crystals (BiFeO_3_ [23], (Pb_0.97_La_0.03_) (Zr_0.52_Ti_0.48_)O_3_ (PLZT) [22], BaTiO_3_ [24]) or thin films (BaTiO_3_ [9], [KNbO_3_]_1–*x*_[BaNi_1/2_Nb_1/2_O_3–δ_]*_x_* [25]). However, there is a small number of papers in this field relevant to nanosized ferroelectric layers, i.e., PLZT [26], Pb(Zr_20_Ti_80_)O_3_ decorated with Ag nanoparticles [20], Si doped HfO_2_ [27], Bi_4_Ti_3_O_12_ [19], Sb-doped ZnO [28], and BiFeO_3_ [6,15,16,29]. In the case of ferroelectric one-dimensional materials, the FE-PV effect has been described so far only for nanofibers [30] and nanotubes [31] of BiFeO_3_.

The first reports on photovoltaic effect in ferroelectric bulk crystals of antimony sulfoiodide (SbSI) were published in the early 80s of the 20th century [32,33]. In recent years, SbSI has been recognized as a potential earth-abundant absorber for solar cells [12,34]. Nie et al. [35] have reported a fabrication method of a light harvester utilizing SbSI, mesoporous TiO_2_, and asymmetric electrodes (Au/fluorine doped tin oxide (FTO)). A thin film of SbSI has been prepared by solution processing, followed by thermal annealing leading to a reaction between antimony trisulfide (Sb_2_S_3_) and antimony triiodide (SbI_3_). A similar approach for preparation of a solar cell based on a thin film of SbSI has been proposed by Choi et al. [36]. It consists of two steps—the formation of amorphous Sb_2_S_3_ and its transformation to SbSI. However, the methods mentioned above have required complex chemical processing and use of hazardous reagents. Ogawa and coworkers have investigated SbSI bulk crystals for application in different types of photovoltaic devices [37,38]. They have revisited the zero-bias photocurrent in a ferroelectric semiconductor SbSI to unveil nonequilibrium electron transport by the shift current [39,40,41].

In this paper, optical and electrical properties of SbSI nanowires are examined confirming their band gap in the visible wavelength and the ferroelectric transition near room temperature. A ferroelectric-photovoltaic effect in SbSI nanowires is presented for the first time. A novel SbSI-based photovoltaic device has been developed with a new configuration of nanowires/IDEs/substrate, where IDEs refers to interdigitated electrodes. It is significantly different from standard photovoltaic devices that have a common planar or parallel-capacitor type of structure. In this study, it is shown that short-circuit photocurrent and open-circuit photovoltage in the constructed FE-PV device can be switched by applying a poling electric field and they depend on light intensity.

## 2. Materials and Methods

SbSI nanowires were prepared via a sonochemical method [42] from the constituents, i.e., the elements—antimony, sulfur, and iodine weighed in the stoichiometric ratio. The component mixture was immersed at room temperature and ambient pressure in ethanol. The reagents were put into a polyethylene/polypropylene cylinder. The vessel was closed during the experiment to prevent volatilization of the precipitant during the longer test times. The cylinder was partly submerged in water in a cup-horn ultrasonic reactor (750 Watt ultrasonic processor VCX-750 with a sealed VC-334 converter (Sonics & Materials, Inc., Newtown, CT, USA)). The reagents were irradiated by ultrasounds with 20 kHz frequency and 565 W/cm^2^ power density for 2 h. The temperature of sonolysis was 323 K. Further experimental details of the applied procedure are described in previous works [42,43]. When the sonochemical process was completed, the ethanol was evaporated off giving a SbSI xerogel.

The structure of individual SbSI nanowire was analyzed using a high-resolution transmission electron microscopy (HRTEM). These investigations were completed at 300 kV accelerating voltage on a JEOL-JEM 3010 microscope (Peabody, MA, USA) with point-to-point resolution of 0.17 nm. The procedure of sample preparation was the same as described in [42,43]. The morphology of aligned SbSI nanowires was studied at acceleration voltage of 10 kV on Phenom Pro X (Thermo Fisher Scientific, Waltham, MA, USA) scanning electron microscope (SEM).

The optical diffuse reflection spectroscopy (DRS) was carried out using the apparatus described in [42]. DRS spectra were recorded at 296 K in the wavelength range from 350 to 1000 nm. The diffuse reflectance values *R*_d_ were converted to the Kubelka–Munk function (*F*_K–M_) [44], known to be proportional to the absorption coefficient α.

The procedure of the FE-PV device preparation can be summarized as follows. Alumina chip #103 (Electronics Design Center, Case Western Reserve University, Cleveland, OH, USA) was used as substrate. It was equipped with platinum interdigitated electrodes separated by a gap of 250 μm. A substrate with platinum electrodes was chosen due to the fact that the energy level of the Pt electrode is close to Fermi energy in the p-type SbSI semiconductor [45,46], which should influence a relatively high zero-bias photocurrent [38]. Symmetric electrodes were used to eliminate the effects of different work functions and asymmetric Schottky-Ohmic contacts on the photovoltaic properties of SbSI. In the first step of device preparation, SbSI xerogel was dispersed uniformly in toluene using the ultrasonic reactor IS-UZP-2 (InterSonic, Olsztyn, Poland). Then a droplet of dispersed solution was placed onto the #103 chip using an insulin syringe equipped with a 31 G needle. The direct current (DC) electric field of 5∙× 10^5^ V/m was applied to the electrodes during the deposition of SbSI sol in order to align the nanowires perpendicularly to the electrodes. Each single coating process was followed by sample drying for toluene evaporation. It was realized at room temperature for 5 min in 830-ABC/EXP glove box (Plas-Labs Products). The steps from sol deposition to sample drying were repeated 15 times. The SbSI FE-PV device, prepared according to the procedure mentioned above, had lateral architecture, in which a photocurrent could be measured along the polarization direction.

Dark current and the photocurrent were detected by a Keithley 6430 Sub-Femtoamp Remote SourceMeter equipped with a low noise probe station (Tektronix, Inc., Beaverton, OR, USA). The acquisition of the data was realized using a PC computer with a GPIB (General Purpose Interface Bus) and an appropriate program in LabView (National Instruments, Austin, TX, USA). All electrical measurements were performed in a test chamber in a vacuum (*p* = 2·× 10^−3^ Pa) produced by TW70H turbomolecular vacuum pump (Prevac, Rogow, Poland) in order to eliminate the influence of humidity [47] and gas adsorption [2,7] on electrical properties of SbSI nanowires. A constant operating temperature *T* = 268 K of the SbSI FE-PV device was maintained using a HAAKE DC30 thermostat with a Kessel HAAKE K20 circulator (Thermo Scientific, Waltham, MA, USA), and Pt-100 sensor with 211 temperature controller (Lake Shore, Columbus, OH, USA). Measurements of photocurrent were carried out under illumination with monochromatic light (λ = 488 nm) from Reliant 50 s argon laser (Laser Physics, Milton Green, UK), which covered the whole sample area between the electrodes. The neutral filters UV–NIR-FILTER-250–2000 nm (Quartzglas-Substrate, Oriel) were applied to change the optical power density (*P*_opt_). The values of *P*_opt_ were determined using S-2387 silicon photodiode (Hamamatsu, Hamamatsu City, Japan) in a short-circuit regime with a Keithley 6517A electrometer. Prior to the photocurrent measurements, the SbSI FE-PV device was poled by cooling from 320 to 268 K under an external electric field of ±10^6^ V/m to align the polarization in one direction, hereafter indicated as positive (+*P*) or negative poling (−*P*).

## 3. Results

**Figure 1:**
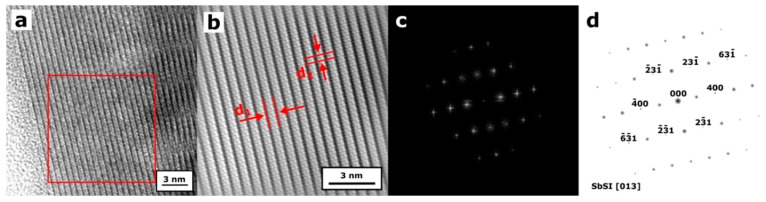
(**a**) Typical high-resolution transmission electron microscopy (HRTEM) micrograph of an individual SbSI nanowire from sonochemically prepared xerogel; (**b**) selected area of HRTEM micrograph marked in (**a**) filtered using fast Fourier transform (FFT) image processing; (**c**) electron diffraction pattern of single SbSI nanowire in the orientation close to the [013] zone axis and (**d**) its simulated diagram; the fringe spacings of *d*_1_ = 0.649 (5) nm and *d*_2_ = 0.414 (4) nm correspond to the interplanar distances of 0.64989 and 0.4160 nm between the (110) and (001) planes of SbSI crystal [48], respectively.

**Figure 2:**
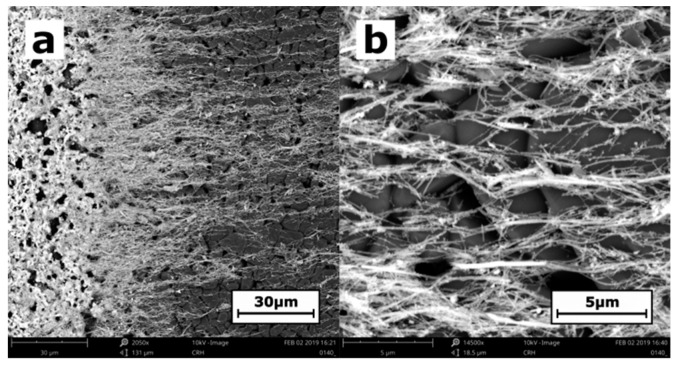
Typical SEM micrograph of aligned SbSI nanowires (**a**) near Pt electrode (left side) and (**b**) between electrodes on Al_2_O_3_ substrate.

The clear lattice fringes in the HRTEM image, as shown in Figure 1a,b, of an individual SbSI nanowire confirm a good single-crystal structure of this material. The fringe spacings of *d*_1_ = 0.649 (5) nm and *d*_2_ = 0.414 (4) nm correspond to the interplanar distances of 0.64989 and 0.4160 nm between the (110) and (001) planes in the Pnam structure of SbSI crystal [48], respectively. The selected area electron diffraction (SAED) pattern, shown in Figure 1c,d, is appropriate for the orthorhombic structure of bulk SbSI crystals. Figure 2 presents a typical SEM micrograph of aligned SbSI nanowires deposited on alumina substrate with platinum electrodes. Most of the nanowires are oriented in a direction perpendicular to the electrodes.

**Figure 3:**
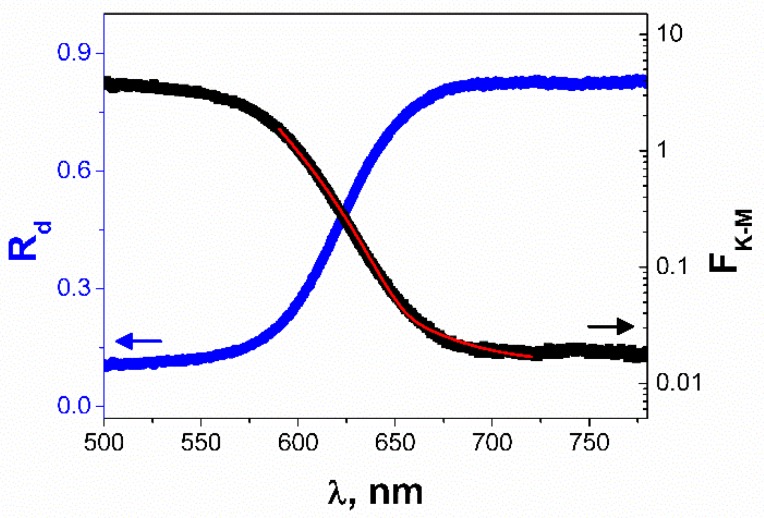
Diffuse reflectance spectrum (●) and the calculated spectrum of Kubelka–Munk function (■) of SbSI gel. The red solid curve represents the fitted theoretical dependence (1) for the sum of indirect forbidden absorption without excitons and phonon statistics, Urbach ruled absorption, and constant absorption term (*T* = 296 K; values of the fitted parameters are given in Table 1).

Figure 3 shows the diffuse reflectance spectrum and the least square fitting of the Kubelka–Munk function derived from the measured diffuse reflectance of SbSI gel. These data allow for the determination of the optical energy gap of the semiconductor using the method described in [49]. The red solid curve in Figure 3 represents the fitted theoretical dependence for the sum (α_t_) of indirect forbidden absorption without excitons and phonon statistics (α_1_), Urbach ruled absorption (α_2_), and constant absorption term (α_3_).

(1) $$\alpha_{t}=\alpha_{1}+\alpha_{2}+\alpha_{3}$$

where

(1a) $$\alpha_{1}=\{\begin{array}{c} 0\;\mathrm{for}\;h\nu \leq E_{\mathrm{gIf}}\\ A_{60}{\left(h\nu -E_{\mathrm{gIf}}\right)}^{3}\;\mathrm{for}\;h\nu >E_{\mathrm{gIf}} \end{array}$$

(1b) $$\alpha_{2}=A_{U}\;\exp\left(\frac{h\nu }{E_{U}}\right)$$

(1c) $$\alpha_{3}=A_{0}.$$

*E*_gIf_ represents the indirect forbidden energy gap, *E*_U_ is the Urbach energy, A_0_, A_60_, A_U_ are constant parameters. The attenuation coefficient A_0_ is the sum of the scattering and absorption independent of the radiation wavelength near the absorption edge. The determined values of the fitted parameters are given in Table 1.

**Figure 4:**
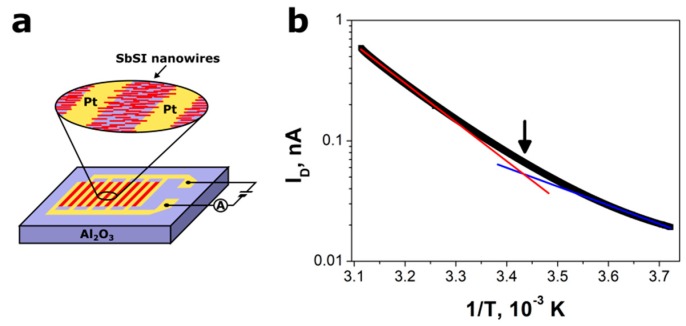
(**a**) A schematic representation of prepared Pt/SbSI/Pt device and (**b**) temperature dependence of electric current flowing through SbSI nanowires (*E* = 10^6^ V/m; *p* = 10^−3^ Pa, RH = 0%); blue and red lines represent the fitted Equation (2) in ferroelectric and paraelectric phases, respectively; the arrow shows Curie temperature; values of fitted parameters are given in Table 2.

Temperature dependence *I*_D_ (T) of dark current flowing through aligned SbSI nanowires, as shown in Figure 4a, is depicted in Figure 4b. The presented results (obtained for heating of the FE-PV device) were practically equal to the ones measured for cooling. These experiments prove semiconductor properties of the investigated material. As for typical ferroelectric, the slope of *I*_D_ (T) changes near the temperature of phase transition (*T*_C_). The temperature dependence of dark current was least squares fitted in the paraelectric and ferroelectric regions using the following theoretical relation:

(2) $$I_{D}\left(T\right)=I_{0}\cdot \exp\left(-\frac{E_{A}}{k_{B}T}\right)$$

where *E*_A_ represents the activation energy, *I*_0_ is the proportionality factor, *k*_B_ and *T* have their usual meanings. The values of fitted parameters *E*_A_ and *I*_0_ are given in Table 2. The intersection of straight-line extrapolations below and above the knee of the *I*_D_ (*T*) characteristic, as shown in Figure 4b, was used to determine Curie temperature *T*_C_ = 291 (2) K.

**Figure 5:**
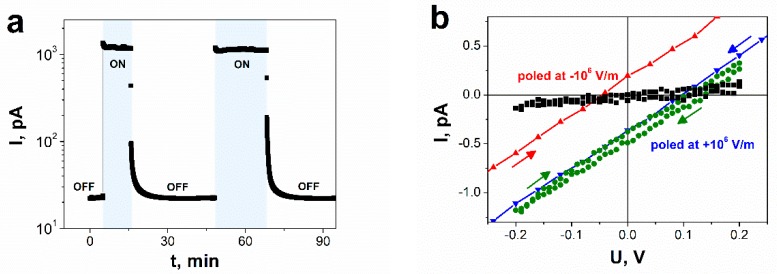
(**a**) A transient response of electric current flowing through SbSI nanowires at a constant bias voltage of 125 V (*E* = 5∙× 10^5^ V/m) in the darkness (OFF) under illumination of Ar laser (ON); (**b**) current–voltage characteristics of the polarity-switchable SbSI ferroelectric-photovoltaic (FE-PV) device in the darkness (■) and under illumination after application of −10^6^ V/m (▲) and +10^6^ V/m (▼,●) poling electric field (λ = 488 nm; *P*_opt_ = 127 mW/cm^2^; *T* = 268 K; *p* = 2·× 10^−3^ Pa; arrows indicate the forward/reverse scan).

To further characterize the photosensitivity of aligned SbSI nanowires, transient studies under constant bias voltage of 125 V were carried out, as shown in Figure 5a. The SbSI FE-PV device was periodically exposed to visible light illumination (λ = 488 nm) and the corresponding photocurrent flow was recorded. The device was found to have a fast response from the OFF to ON state with time constant less than 0.84 s. The relative photoconductivity (RPC) can be defined as follows [50]:

(3) $$\gamma =\frac{I_{\mathrm{ph}}-I_{d}}{I_{d}}$$

where *I*_ph_ and *I*_d_ are electric currents measured under illumination and in the darkness, respectively. A significant RPC effect was observed for SbSI nanowires (γ = 51.6), which is nearly equal to γ ≈ 50 determined for a single nanowire of ZnO at a temperature of 300 K [50].

Current–voltage characteristics of the FE-PV SbSI device in the darkness and under laser irradiation (λ = 488 nm) are shown in Figure 5b. The measurements were carried out after illuminating the sample for 10 min to eliminate the current origin from the transient sample temperature change. Before the experiment, the device was poled by applying a high external electric field of 10^6^ V/m. The forward scan is defined here as the voltage decrease from positive to negative values, whereas reverse scan refers to a voltage change in the opposite direction. Interestingly, a linear forward scan from +0.2 to −0.2 V has a similar performance as a reverse scan in the same range of bias voltage. These results imply that applying a bias voltage of 0.2 V is not sufficient to reverse a polarization direction. When a higher preset voltage is used (red and blue curves in Figure 5b), current-voltage characteristics exhibit hysteresis.

The *I*–*V* curves under illumination show photovoltaic behavior with opposite signs of short-circuit photocurrent (*I*_SC_) and open-circuit photovoltage (*V*_OC_) against the origin, depending on the voltage sweep history. In the case of the forward scan, the values of *I*_SC_ and *V*_OC_ are about 0.36 pA and 97 mV, respectively. The short-circuit current of 0.20 pA and open-circuit voltage of 45 mV were estimated for a reverse scan.

**Figure 6:**
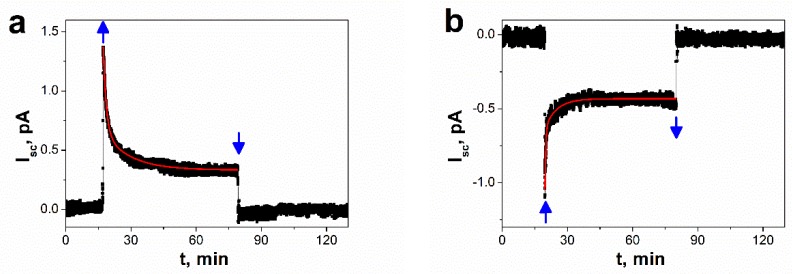
Time dependences of short-circuit photovoltaic current responses on switching on (↑) and switching off (↓) illumination of the SbSI FE-PV device with different poling electric fields: (**a**) *E* = −10^6^ V/m, (**b**) *E* = +10^6^ V/m (λ = 488 nm; *P*_opt_ = 127 mW/cm^2^; *U* = 0 V; *T* = 268 K; *p* = 2 × 10^−3^ Pa; red solid curves represent the best fitted dependences described by Equation (4); values of the fitted parameters are presented in Table 3).

Figure 6 illustrates the time dependence of the zero-bias photocurrent. After switching illumination on, the electric photocurrent increases fast, attains maximum, and then slowly decreases with time to a stationary value. *I*_SC_(t) responses of SbSI nanowires, as shown in Figure 6, were least square fitted with an empirical formula

(4) $$I_{\mathrm{sc}}\left(t\right)=I_{s}+I_{1}e^{-\left(t-t_{\mathrm{on}}\right)/\tau_{1}}+I_{2}e^{-\left(t-t_{\mathrm{on}}\right)/\tau_{2}},\;\mathrm{for}\;t\geq t_{\mathrm{on}}$$

where *t*_on_ represents time when the illumination was switched on; *I*_S_ is the stationary value of short-circuit photocurrent; *I*_1_, *I*_2_ are the pre-exponential factors; τ_1_ and τ_2_ are the time constants. Values of the fitted parameters are presented in Table 3. A transient photocurrent with relaxation time τ_1_ can be ascribed to surface recombination of photogenerated electrons and holes. Whereas the subsequent decay towards the steady state current (τ_2_) results probably from a capture of built-up electrons in surface states inducing holes flux associated with recombination. It should be underlined that the curve of time evolution of short-circuit current has exactly the same shape as *I*_SC_ (*t*) dependences reported for photovoltaic devices based on other ferroelectric materials [16,20,22,24,25,37].

One can see that for the negatively poled FE-PV device the photocurrent is positive, as shown in Figure 6a. In contrast, after the positive poling, the photocurrent direction is reversed, as shown in Figure 6b, and its magnitude is slightly increased. It is characteristic for the FE-PV effect, that the direction of the photocurrent is opposite to the polarization vector [10,15].

**Figure 7:**
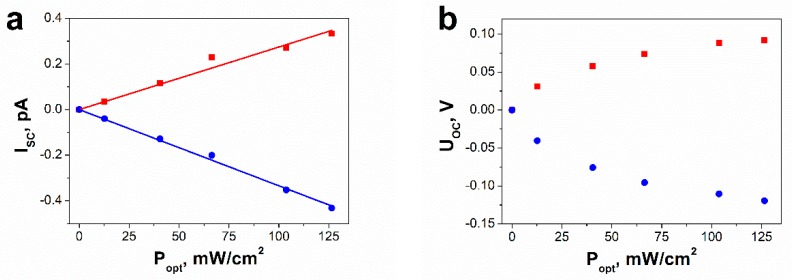
Influence of the optical power density on (**a**) short-circuit photocurrent and (**b**) open-circuit photovoltage of the SbSI FE-PV device with different poling electric fields: (■) *E* = −10^6^ V/m, (●) *E* = +10^6^ V/m (λ = 488 nm; *T* = 268 K; *p* = 2·10^−3^ Pa; solid curves represent the best fitted dependences described by Equation (6); values of the fitted parameters are given in the text).

The influence of the optical power density (*P*_opt_) on the short-circuit photocurrent is presented in Figure 7a. As expected [15], *I*_SC_ is observed to increase almost linearly with the illumination intensity. It is well known that the short-circuit current density due to the bulk-photovoltaic effect under monochromatic illumination is described by an empirical equation called Glass law [10,51]:

(5) $$J_{\mathrm{SC}}=\kappa \alpha P_{\mathrm{opt}}$$

where α is the absorption coefficient, *P*_opt_ represents the optical power density expressed in W/cm^2^, and κ is the Glass constant, which is related to the charge generation and collection efficiency [51]. Glass law (5) can be easily transformed into the following relation:

(6) $$I_{\mathrm{SC}}=A\cdot P_{\mathrm{opt}}$$

where *A* = κ·α·S is linear coefficient dependent on an area of sample cross section S. The experimental data, presented in Figure 7a, were last square fitted with Equation (6). Determined values of *A* coefficient are 2.7 (1)·10^−16^ m^2^/V and −3.34 (6)·10^−16^ m^2^/V in the case of negative and positive poling, respectively.

The open-circuit voltage, corresponding to the condition of *I* = 0, could not be measured due to very high resistance of the SbSI FE-PV device. Thus, values of *V*_OC_ were calculated using the simple relation [10,24,33,51]

(7) $$V_{\mathrm{OC}}=\frac{J_{\mathrm{SC}}}{\sigma_{d}+\sigma_{\mathrm{ph}}}d=R_{\mathrm{IL}}\cdot I_{SC}$$

where *J*_SC_ represents steady short-circuit current density, *d* is distance between electrodes, σ_d_ and σ_ph_ are the dark conductivity and photoconductivity, respectively. *R*_IL_ means electric resistance of SbSI nanowires under illumination, which was determined from current–voltage characteristics. The optical power density dependence of open-circuit photovoltage is given in Figure 7b. *V*_OC_ rises with an increase of light intensity and saturates for high *P*_opt_. The same behavior of *V*_OC_ (*P*_opt_) dependence was also observed in the case of FE-PV devices based on other ferroelectric materials [6,15,51]. The optical power density dependence of the generated photovoltaic voltage is discussed in detail in the Supplementary Materials. At the highest optical power density, the magnitude of *V*_OC_ in the positively poled SbSI nanowires reaches 0.119 (2) V.

## 4. Discussion

The calculated value of *E*_gIf_ = 1.862 (1) eV is very close to the indirect forbidden energy gap of SbSI nanowires reported in the literature for the same technology of material preparation [43,52,53]. The magnitude of band gap is one of the key factors for harvesting visible light energy through the photovoltaic effect [23]. Determined *E*_gIf_ for SbSI nanowires is relatively narrow in comparison to wide band gaps of ferroelectric oxides, that exhibit the vanishingly small photoresponse under visible light [14]. The band gaps of commonly used ferroelectric materials (LiNbO_3_, BaTiO_3_, and PZT) exceed 3 eV. Therefore, they are able to harvest sunlight in the UV range, which constitutes only about 3.5% of total solar energy [51].

Determined Curie temperature *T*_C_ = 291 (2) K is in good agreement with *T*_C_ = 292 (1) K [54] and *T*_C_ = 293.0 (2) K [42] measured for SbSI gel and it is slightly lower than Curie temperature of bulk SbSI crystals [55,56].

One can notice that the thermal activation energies of electrical conductivity determined for SbSI nanowires in ferroelectric and paraelectric phases are different, as shown in Table 2. Unfortunately, even the values of E_A_ reported for bulk SbSI crystals vary in very wide ranges (e.g., from 0.01 to 1.6 eV [57]). Therefore, it is difficult to find the interpretation of E_A_ determined in the case of SbSI nanowires.

The SbSI FE-PV device can be considered as a metal–ferroelectric–metal (MFM) junction with symmetric metal contacts having the same work function. Lopez-Varo and coworkers [10] presented numerical simulation of photoelectrical properties of symmetric-ohmic and symmetric-Schottky MFM diodes. They found that both of these structures under illumination reveal a noticeable hysteresis loop in the *J*–*V* curves similar to characteristics depicted in Figure 5b. The same nature of the *I*–*V* curve, that varies depending on the scan direction and range of the applied voltage during measurements, was reported also for FE-PV devices based on other ferroelectric materials (halide perovskites [58,59,60] and Nd-doped BiFeO_3_ [61]). Such hysteretic behavior can be attributed to existence of interface charge that originates from mobile ions in ferroelectric material [58,60]. This hypothesis should be verified in future investigations of electrical conductivity of SbSI nanowires.

The results shown in Figure 5b confirm a polarization-induced photovoltaic effect in SbSI nanowires. However, a typical response of the FE-PV cell has switchable and unswitchable components [61]. The first one originates from a polarization-induced field, while the latter component is related to a persistent built-in electric field (e.g., Schottky barrier). The magnitudes of these components can be calculated using the following equations [61]:

(8) $$I_{\mathrm{sc}}^{p}=\frac{1}{2}\left|I_{\mathrm{sc}}^{F}-I_{\mathrm{sc}}^{R}\right|,\;I_{\mathrm{sc}}^{bi}=\frac{1}{2}\left|I_{\mathrm{sc}}^{F}+I_{\mathrm{sc}}^{R}\right|$$

(9) $$V_{\mathrm{oc}}^{p}=\frac{1}{2}\left|V_{\mathrm{oc}}^{F}-V_{\mathrm{oc}}^{R}\right|,\;V_{\mathrm{oc}}^{bi}=\frac{1}{2}\left|V_{\mathrm{oc}}^{F}+V_{\mathrm{oc}}^{R}\right|$$

where superscripts “*F*” and “*R*” refer to the forward sweep and reverse sweep, respectively, symbols “*p*” and “*bi*” denote the polarization-induced and built-in contribution to *I*_SC_/*V*_OC_, respectively. The values of the aforementioned components $I_{\mathrm{sc}}^{p}$ = 0.28 pA, $I_{\mathrm{sc}}^{bi}$ = 0.08 pA, $V_{\mathrm{oc}}^{p}$ = 71 mV, $V_{\mathrm{oc}}^{bi}$ = 26 mV were calculated from *I*-*V* characteristics, as shown in Figure 5b. One can notice that the switchable component of the photovoltaic response of SbSI nanowires prevails.

**Figure 8:**
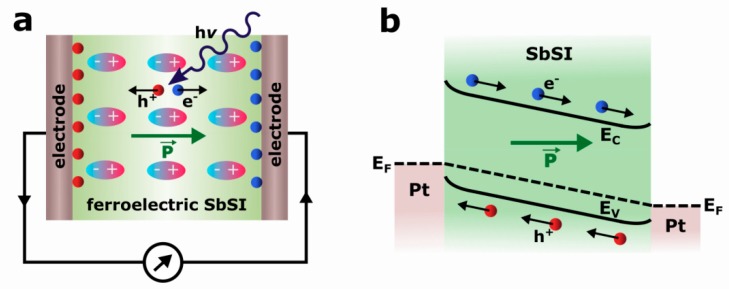
The mechanism of photocurrent generation (**a**) and energy band diagram (**b**) of a poled Pt/SbSI/Pt ferroelectric-photovoltaic device (e^−^—electron; h^+^—hole; P—electric polarization; hν—photon; *E*_F_—Fermi level; *E*_C_—bottom of conduction energy band; *E*_V_—top of valence energy band).

The mechanism of the FE-PV effect in poled Pt/SbSI/Pt device, as shown in Figure 8a, can be explained as follows. MFM structure with symmetric metal contacts is expected to yield a symmetric photovoltaic diode performance [11]. The same Schottky barriers are present on both sides of the Pt/SbSI/Pt device, as shown in Figure 8b. The band bending in a semiconductor in contact with a metal electrode can generate classical photocurrents via the drift-diffusion process [38]. They flow in opposite directions and cancel each other. Thus, the photovoltaic effect in SbSI nanowires is not due to the presence of the depletion layer in the metal/ferroelectric interface with Schottky contact. Moreover, the relatively large distance between electrodes (250 μm) eliminates the Schottky barrier effect on the photovoltaic performance in the Pt/SbSI/Pt device [13]. The photovoltaic mechanism of the presented device can be proposed as a bulk photovoltaic effect. When the light with an energy higher than the band gap of ferroelectric is absorbed, the excess carriers (electrons and holes) are photogenerated, as shown in Figure 8a. A spontaneous polarization in poled ferroelectric SbSI leads to band bending, as shown in Figure 8b, and it is responsible for an existence of internal electric field along the c-axis of nanowires. Consequently, excess carriers are driven in opposite directions toward electrodes and contribute to the photovoltaic output, as shown in Figure 8a. One can see that the photocurrent is generated only in the purely SbSI bulk region. It should be underlined that an internal electric field originating from spontaneous polarization is frequently demonstrated as a key factor determining a spatial separation of charge carriers in FE-PV devices [13,15,21,22,31,62,63].

Antimony sulfoiodide belongs to group of crystals with noncentrosymmetric structure [38,64]. An origin of a bulk photovoltaic effect in SbSI is usually ascribed to the shift current [38,39,40,41], which is recognized as a result of the second-order nonlinear optical response [38]. The form of the response function implies that the position of the electron wave packet immediately shifts in real space upon the interband optical transition [41]. A shift vector [65], recognized as an average distance and direction of the shift, is given by the difference in the Berry connection of the Bloch wave functions [40,41] of the two corresponding bands. It has a non-zero value only when the inversion symmetry is broken. The shift vector is closely related to electric polarization [38,41]. The spontaneous polarization of a ferroelectric consists of the ionic (*P*_ion_) and the electronic (*P*_el_) constituents [66]. The first one arises from the displacement of charged atoms or molecules, whereas the latter comes from the asymmetry in the wave function forming the covalent bonds [41]. According to a recent theory of polarization, the Berry phase [66] for the valence states can be applied for determination of the electronic component of the spontaneous polarization. The shift vector is also expressed using the geometric phase. Its form indicates the change in *P*_el_ induced by the optical transition between the conduction and valence band [41]. Former studies of an origin of ferroelectricity in SbSI [67] ascribed a net dipole moment per unit volume, or a polarization to small displacement of Sb relative to S and I atoms. The switchable nature of the photocurrent, as shown in Figure 5b, Figure 6, and Figure 7a, and photovoltage, as shown in Figure 5b and Figure 7b, by poling SbSI nanowires along the two different directions is direct evidence that the ferroelectric polarization plays a crucial role in the observed bulk photovoltaic effect.

**Figure 9:**
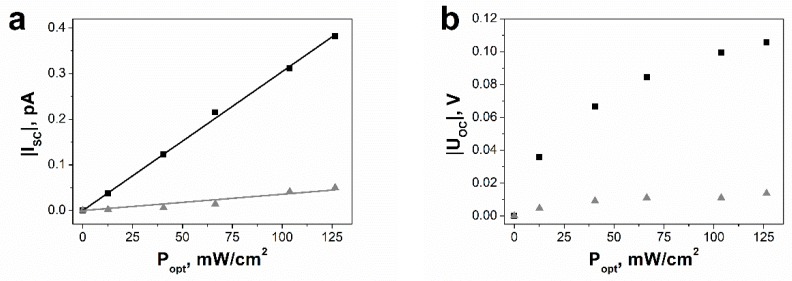
Influence of the optical power density on separated contributions to (**a**) *I*_SC_ and (**b**) *V*_OC_ from the switchable polarization (■) and unswitchable internal bias field (▲) in the SbSI FE-PV device (λ = 488 nm; *T* = 268 K; *p* = 2·10^−3^ Pa; solid curves represent the best fitted dependences described by Equation (6); values of the fitted parameters are given in the text).

The photovoltaic responses of the SbSI FE-PV device to illumination with different optical power densities, as shown in Figure 7, can be separated into two independent components [11,15],

(10) $$I_{\mathrm{sc}}^{p}=\frac{1}{2}\left|I_{\mathrm{sc}}^{+}-I_{\mathrm{sc}}^{-}\right|,\;I_{\mathrm{sc}}^{bi}=\frac{1}{2}\left|I_{\mathrm{sc}}^{+}+I_{\mathrm{sc}}^{-}\right|$$

(11) $$V_{\mathrm{oc}}^{p}=\frac{1}{2}\left|V_{\mathrm{oc}}^{+}-V_{\mathrm{oc}}^{-}\right|,\;V_{\mathrm{oc}}^{bi}=\frac{1}{2}\left|V_{\mathrm{oc}}^{+}+V_{\mathrm{oc}}^{-}\right|$$

where $I_{\mathrm{sc}}^{p}$, $V_{\mathrm{oc}}^{p}$ are contributions originating from the switchable ferroelectric polarization, $I_{\mathrm{sc}}^{bi}$, $V_{\mathrm{oc}}^{bi}$ are components induced by the unswitchable internal bias field, superscripts “+” and “−” refer to the positive and negative poling, respectively. The results of this deconvolution are plotted in Figure 9. Black and grey lines in Figure 9a represent least square fitting the experimental data with Glass law (6). Determined values of the A coefficient are 3.04 (3)·10^−16^ m^2^/V and 3.6 (4)·10^−17^ m^2^/V in the case of separated components from the switchable polarization and unswitchable internal bias field, respectively. The contribution to the short-circuit current coming from the switchable polarization is about 8.5 times larger than that from the unswitchable internal field at the highest value of optical power density, as shown in Figure 9a. This ratio for open-circuit voltage is 7.7, as shown in Figure 9b. The existence of a weak built-in electric field in the SbSI FE-PV device can be explained taking into account two probable effects—presence of iodine vacancies or other defects [15,23] as well as nonuniform distribution of SbSI nanowires [51].

Determination of short-circuit photocurrent density in the SbSI FE-PV device is complicated due to imperfect alignment and distribution of SbSI nanowires, as shown in Figure 2. Nevertheless, the minimum value of current density *J*_SCmin_ = 7.4 nA/cm^2^ for *P*_opt_ = 127 mW/cm^2^ was estimated, taking into account geometrical parameters of IDEs on #103 substrate, the average lateral dimension of SbSI nanowire *d* = 30 nm [42,43], and assuming that the area between electrodes is fully covered by SbSI nanowires. One can expect that the real value of *J*_SC_ is much higher than evaluated *J*_SCmin_.

The open-circuit photovoltage determined for SbSI nanowires is comparable to values of *V*_OC_ reported in the literature for other ferroelectric nanomaterials, as shown in Table 4. However, photovoltage can be enlarged in the future by increasing the distance between electrodes. Furthermore, improvement of electrical contacts between separate nanowires should lead to an enhancement of short-circuit photocurrent.

## 5. Conclusions

In summary, SbSI nanowires have been confirmed to be ferroelectric semiconductors with a Curie temperature of *T*_C_ = 291 (2) K and an energy band gap of *E*_gIf_ = 1.862 (1) eV beneficial for their application in photovoltaic devices.

The current–voltage characteristics of the Pt/SbSI/Pt device under illumination show photovoltaic behavior with opposite signs of short-circuit photocurrent and open-circuit photovoltage against the origin, depending on the poling and voltage sweep history. A hysteresis loop observed in the I–V curves under illumination is typical for symmetric metal–ferroelectric–metal structures.

The generation of steady-state photocurrent in SbSI nanowires in the absence of external voltage application has been presented for the first time. The short-circuit photocurrent versus illumination intensity has found to follow Glass law. The large portion of the short-circuit photocurrent and open-circuit photovoltage (of about 89%) is changeable in response to an external poling electric field. This switchable nature of the photoresponse of SbSI nanowires indicates that the ferroelectric polarization plays a dominant role in the observed photovoltaic effect. It should be emphasized that the presence of the depletion layer in the metal/ferroelectric interface with Schottky contact is not responsible for the photovoltaic effect in SbSI nanowires. On the contrary, the mechanism of the presented FE-PV effect device can be proposed as a bulk photovoltaic effect, where a spontaneous polarization results in an existence of internal electric field along the c-axis of nanowires. The photocurrent is generated only in the purely SbSI bulk region and it can be recognized as a shift current.

The open-circuit photovoltage in SbSI nanowires has found to be comparable to values of *V*_OC_ determined for FE-PV devices based on other ferroelectric nanomaterials. It should be underlined that these results have been obtained from a Pt/SbSI/Pt device as proof of concept. Further optimization on electrode materials selection and distance between electrodes should enhance the open-circuit photovoltage. The short-circuit photocurrent can be increased in the future in two ways. The first possible manner refers to the improvement of the electrical contacts between SbSI nanowires. The second method is based on band gap engineering, which includes doping, change of chemical composition, and strain application.

This paper has presented a simple and non-destructive method of determination of the polarization direction (stored information) by sensing the photovoltage or photocurrent in an SbSI FE-PV device. This is clear evidence that ferroelectric SbSI nanowires are promising for application in non-volatile memories based on the photovoltaic effect, where the photovoltaic output is used as the read-out signal. Moreover, one-dimensional nanostructures of SbSI can be considered in the future as suitable candidates for construction of novel self-powered photodetector systems.

## Figures and Tables

**Table 1:** Values of parameters of Equation (1) determined from the fitting of the Kubelka–Munk function of SbSI gel presented in Figure 3.

Fitted Parameters	Values
A_60_, (eV)^−3^m^−1^	81 (2)
*E*_gIf_, eV	1.862 (1)
A_U_, 10^−13^m^−1^	6.4 (6)
*E*_U_, eV	0.078 (3)
A_0_, 10^−3^m^−1^	14.4 (4)

**Table 2:** Parameters of Equation (2) fitted to temperature dependence of dark current flowing through SbSI nanowires, as shown in Figure 4b, in vacuum (*p* = 10^−3^ Pa).

Phase	*E*_A_, eV	*T*_C_, K
ferroelectric	0.3083 (6)	291 (2)
paraelectric	0.6422 (6)	

**Table 3:** Parameters of Equation (4) fitted to short-circuit photocurrent responses of SbSI nanowires to switching on illumination, as shown in Figure 6.

Parameter	Poling Electric Field	
	Positive	Negative
*I*_s_, pA	−0.432 (1)	0.332 (1)
*I*_1_, pA	−0.51 (2)	0.716 (7)
*I*_2_, pA	−0.200 (5)	0.320 (5)
$\tau_{1}$, s	24 (1)	85 (2)
$\tau_{2}$, min	5.5 (2)	12.2 (3)

**Table 4:** A summary of results achieved for photovoltaic devices based on low dimensional ferroelectrics ^1^.

Ferroelectric Nanomaterial	Electrodes	Illumination	*J*_SC_, µA/cm^2^	*U*_OC_, V	Ref.
PLZT film (*d* = 68 nm)	AEs: LSMO and Nb:STO	UV (λ = 356 nm; 0.86 mW/cm^2^)	2.324	0.71	[26]
PZT film with Ag NPs (*d* = 200 nm)	AEs: ITO and Pt	Xenon lamp (100 mW/cm^2^)	110	0.76	[20]
Si doped HfO_2_ film (*d* = 10 nm)	AEs: Si and Au	AM1.5 (10 mW/cm^2^)	~200	~0.2	[27]
BIT film (*d* = 150 nm)	AEs: FTO and Au	AM1.5 (10 mW/cm^2^)	0.18	0.02	[19]
Sb-doped ZnO nanobranched films	AEs: FTO and Pt	UV (63 mW/cm^2^)	0.05	0.087	[28]
BFO film (*d* = 100 nm)	AEs: LSMO and Fe/Pt	Halogen lamp (20 mW/cm^2^)	-	0.21	[6]
BFO film (*d* = 120 nm)	AEs: LSMO and Pt	Halogen lamp (100 mW/cm^2^)	~2	~0.5	[29]
BFO film (*d* = 170 nm)	AEs: ITO and SRO	Xenon lamp (λ = 435 nm; 0.75 mW/cm^2^)	~0.4	0.3	[15]
BFO NFs	SEs: Au IDEs	AM1.5 (10 mW/cm^2^)	~0.31	0.8	[30]
Pr-doped BFO NTs	SEs: Ag	AM1.5 (10 mW/cm^2^)	0.356	0.21	[31]
SbSI NWs	SEs: Pt IDEs	Argon laser (λ = 488 nm; 127 mW/cm^2^)	>0.0074	0.119(2)	this paper

^1^ The used abbreviations are as follows: AEs—asymmetric electrodes; AM1.5—standard solar white-light illumination; BIT—Bi_4_Ti_3_O_12_; BFO—BiFeO_3_; *d*—thickness of a ferroelectric film; FTO—fluorine-doped tin oxide; IDEs—interdigitated electrodes; ITO—indium tin oxide; LSMO—La_0.7_Sr_0.3_MnO_3_; Nb:STO—Nb-doped SrTiO_3_; NFs—nanofibers; NPs—nanoparticles; NWs—nanowires; NTs—nanotubes; PLZT—(Pb_0.97_La_0.03_) (Zr_0.52_Ti_0.48_)O_3_; PZT—Pb(Zr_20_Ti_80_)O_3_; SEs—symmetric electrodes; SRO—SrRuO_3_.

## Supplementary Materials

The following are available online at https://www.mdpi.com/2079-4991/9/4/580/s1, Figure S1: Influence of optical power density on (a) photoconductivity current (b) reciprocal of the electric resistance of SbSI FE-PV device at constant voltage bias of U=1 V. Red solid curves represent the best fitted dependence described by Eq. (S1); Values of the fitted parameters are given in the text; Blue dashed line and green arrow correspond to values of expressions σ_d_∙*S*/*d* and (σ_ph_+σ_d_)*S*/*d*, respectively.
