# Supplementary material for: A Ferroelectric-Photovoltaic Effect in SbSI Nanowires
Source: Nanomaterials (Basel). 2019 Apr 9;9(4):580. doi: 10.3390/nano9040580 (PMC6523164; doi:10.3390/nano9040580)
Supplement: Supplementary file 1 [file nanomaterials-09-00580-s001.pdf]

Supplementary Information for

A ferroelectric photovoltaic effect in SbSI nanowires

Krystian Mistewicz [1],*, Marian Nowak [1] and Danuta Stróż [2]

[1] Institute of Physics – Center for Science and Education, Silesian University of Technology, Krasińskiego 8, 40-019 Katowice, Poland; krystian.mistewicz@polsl.pl, marian.nowak@polsl.pl

[2] Institute of Material Science, University of Silesia, 75 Pułku Piechoty 1A, 41-500 Chorzów, Poland; danuta.stroz@us.edu.pl

* Correspondence: krystian.mistewicz@polsl.pl; Tel.: +48-32-603-4156

In this "Supplementary Information", we present the optical power density dependence of the photoconductivity in Pt/SbSI/Pt device under monochromatic illumination (λ=488 nm). This relation influences the optical power density dependence of the generated photovoltaic voltage.

The photoconductivity (σ_{ph}) as well as photoconductivity current (I_{ph}) of SbSI nanowires is a nonlinear function of the light intensity (P_{opt})

$$I_{ph} = I_{ph0} \cdot P_{opt}^{\gamma}, \qquad \text{(S1)}$$

where I_{ph0} is pre-exponential factor and γ denotes power coefficient. This dependence was confirmed for SbSI nanowires experimentally and published in different papers (see Refs. [S1,S2,S3]).

Influence of optical power density on photoconductivity current flowing through SbSI FE-PV device at constant voltage bias of U=1 V is shown in Figure S1a. Red solid curve represents the best fitted dependence described by Eq. (S1); Values of the fitted parameters are following: γ=0.52(6), I_{ph0}=86(33) fA. The evaluated value of power coefficient indicates the nonlinear recombination of carriers in SbSI nanowires with the increase of excess carrier concentration. It is in agreement with the data published on photoconductivity of nanowires as well as single crystals of SbSI (see Fig. 7 in Ref. [S4]).

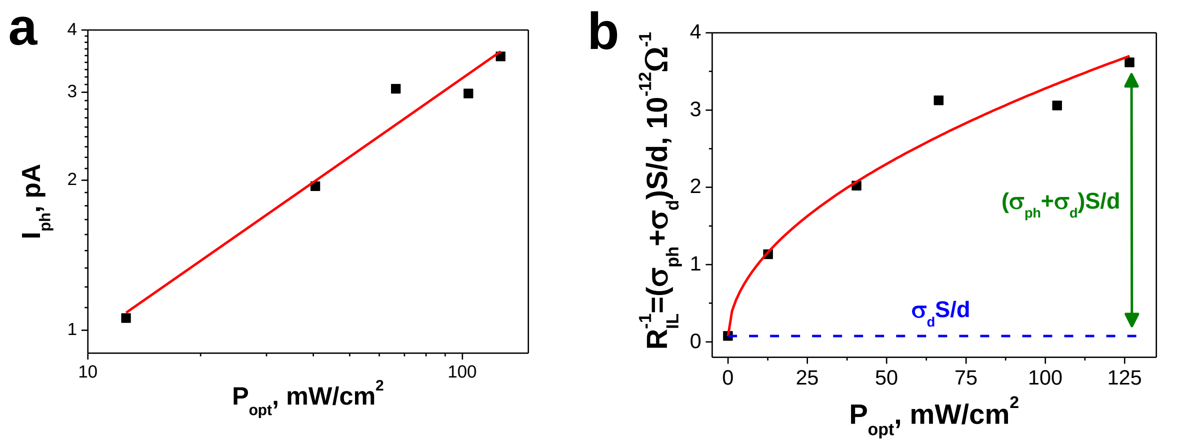

Figure S1. Influence of optical power density on (a) photoconductivity current (b) reciprocal of the electric resistance of SbSI FE-PV device at constant voltage bias of U=1 V. Red solid curves represent the best fitted dependence described by Eq. (S1); Values of the fitted parameters are given in the text; Blue dashed line and green arrow correspond to values of expressions $\sigma_d \cdot S/d$ and $(\sigma_{ph}+\sigma_d)S/d$, respectively.

According to Eq. (7), given in the main paper, the reciprocal of the electric resistance of SbSI nanowires under illumination (R_{IL}) is proportional to total electric conductance, i.e. sum of photoconductivity (σ_{ph}) and dark conductivity (σ_d)

$$R_{IL}^{-1} = \frac{(\sigma_{ph}+\sigma_d)S}{d} \qquad (S2)$$

where d represents electrode distance and S is total cross section of nanowires in SbSI FE-PV device.

One can see in Figure S1b that, total electric conductance of SbSI nanowires is nonlinear function of optical power density. Therefore, according to Eq. (7), provided in the article manuscript, the open circuit voltage (V_{OC}) increases nonlinearly with increasing P_{opt} and it saturates at high P_{opt} (Figs. 7b and 9b in the manuscript).

It should be underlined that, the same character of dependence $V_{OC}(P_{opt})$ was reported for other FE-PV devices, eg. $BiFeO_3$ [S5,S6], Bi_2FeCrO_6 [S7].

References

[S1] Mistewicz, K. Recent Advances in Ferroelectric Nanosensors: Toward Sensitive Detection of Gas, Mechanothermal Signals, and Radiation. *J. Nanomater.* **2018**, *2018*, 1-15, Article ID 2651056. https://doi.org/10.1155/2018/2651056

[S2] Nowak, M.; Mistewicz, K.; Nowrot, A.; Szperlich, P.; Jesionek, M.; Starczewska, A. Transient characteristics and negative photoconductivity of SbSI humidity sensor. *Sens. Actuator A* **2014**, *210*, 32-40. http://dx.doi.org/10.1016/j.sna.2014.02.004

[S3] Nowak, M.; Bober, Ł.; Borkowski, B.; Kępińska, M.; Szperlich, P.; Stróż, D.; Sozańska, M. Quantum efficiency coefficient for photogeneration of carriers in SbSI nanowires. *Opt. Mater.* **2013**, *35*, 2208–2216. http://dx.doi.org/10.1016/j.optmat.2013.06.003

[S4] Nowak, M.; Szperlich, P.; Kidawa, A.; Kepinska, M.; Gorczycki, P.; Kauch, B. Optical and photoelectrical properties of SbSI. *Proc. SPIE* **2003**, *5136*, 172–177. http://dx.doi.org/10.1117/12.518846

[S5] Ji, B.W.; Yao, K.; Liang, Y.C. Bulk Photovoltaic Effect at Visible Wavelength in Epitaxial Ferroelectric $BiFeO_3$ Thin Films. *Adv. Mater.* **2010**, *22*, 1763–1766. https://doi.org/10.1002/adma.200902985

[S6] Guo, R.; You, L.; Zhou, Y.; Lim, Z.S.; Zou, X.; Chen, L.; Ramesh, R.; Wang, J. Non-volatile memory based on the ferroelectric photovoltaic effect. *Nat. Commun.* **2013**, *4*, Article number: 1990. https://doi.org/10.1038/ncomms2990

[S7] Quattropani, A.; Makhort, A.S.; Rastei, M.V.; Versini, G.; Schmerber, G.; Barre, S.; Dinia, A.; Slaoui, A.; Rehspringer, J.-L.; Fix, T.; Colis, S.; Kundys, B. Tuning photovoltaic response in Bi_2FeCrO_6 films by ferroelectric poling. *Nanoscale* **2018**, *10*, 13761. https://doi.org/10.1039/C8NR03137A
